# Supplementary material for: Hot spot prediction in protein-protein interactions by an ensemble system
Source: BMC Syst Biol. 2018 Dec 31;12(Suppl 9):132. doi: 10.1186/s12918-018-0665-8 (PMC6311905; doi:10.1186/s12918-018-0665-8)
Supplement: Supplementary file 2 — All relevant MATLAB code. A simple Matlab implement of our predictor. (ZIP 19,742 kb) [file 12918_2018_665_MOESM2_ESM.zip › Additional file 2/Read ASA/ASA website/dbMPIKT/4J2LC.html]

 NetSurfP - Protein Surface Accessibility and Secondary Structure Predictions

  

|  |  |  |
| --- | --- | --- |
|  |  | NetSurfP - Protein Surface Accessibility and Secondary Structure Predictions  Technical University of Denmark |

  


---

```
# For publication of results, please cite:
# A generic method for assignment of reliability scores applied to solvent accessibility predictions.
# Bent Petersen, Thomas Nordahl Petersen, Pernille Andersen, Morten Nielsen and Claus Lundegaard
# BMC Structural Biology 2009, 9:51 doi:10.1186/1472-6807-9-51
#
# Column 1: Class assignment - B for buried or E for Exposed - Threshold: 25% exposure, but not based on RSA
# Column 2: Amino acid
# Column 3: Sequence name
# Column 4: Amino acid number
# Column 5: Relative Surface Accessibility - RSA
# Column 6: Absolute Surface Accessibility
# Column 7: Z-fit score for RSA prediction
# Column 8: Probability for Alpha-Helix
# Column 9: Probability for Beta-strand
# Column 10: Probability for Coil
E M  4J2LC                  1    0.689 137.889  -0.052   0.003   0.003   0.994
B F  4J2LC                  2    0.271  54.470  -0.970   0.022   0.359   0.619
E V  4J2LC                  3    0.331  50.875  -0.506   0.022   0.552   0.426
B W  4J2LC                  4    0.165  39.658  -0.839   0.022   0.552   0.426
E T  4J2LC                  5    0.433  60.071  -0.794   0.021   0.451   0.528
E N  4J2LC                  6    0.534  78.134  -0.727   0.021   0.279   0.699
B V  4J2LC                  7    0.174  26.713  -0.180   0.020   0.205   0.775
E E  4J2LC                  8    0.543  94.862   0.115   0.019   0.141   0.840
E P  4J2LC                  9    0.358  50.857  -1.103   0.018   0.088   0.893
E R  4J2LC                 10    0.477 109.210  -0.431   0.018   0.088   0.893
E S  4J2LC                 11    0.454  53.221  -0.702   0.021   0.279   0.699
B V  4J2LC                 12    0.145  22.333   0.536   0.021   0.451   0.528
B A  4J2LC                 13    0.248  27.363  -0.864   0.022   0.552   0.426
B V  4J2LC                 14    0.155  23.870  -0.892   0.021   0.451   0.528
B F  4J2LC                 15    0.100  19.990   0.659   0.022   0.359   0.619
B P  4J2LC                 16    0.242  34.397  -0.363   0.020   0.205   0.775
B W  4J2LC                 17    0.106  25.421  -0.427   0.056   0.142   0.802
B H  4J2LC                 18    0.255  46.312  -0.623   0.056   0.142   0.802
E S  4J2LC                 19    0.306  35.863  -1.091   0.118   0.150   0.732
B L  4J2LC                 20    0.093  17.010  -0.256   0.066   0.296   0.638
B V  4J2LC                 21    0.087  13.403   0.845   0.064   0.216   0.721
B P  4J2LC                 22    0.267  37.944  -0.463   0.064   0.216   0.721
B F  4J2LC                 23    0.200  40.040  -0.985   0.066   0.296   0.638
B L  4J2LC                 24    0.190  34.862  -1.027   0.021   0.279   0.699
B A  4J2LC                 25    0.259  28.586  -0.551   0.020   0.205   0.775
E P  4J2LC                 26    0.392  55.653  -1.275   0.018   0.088   0.893
E S  4J2LC                 27    0.644  75.489  -1.991   0.018   0.047   0.935
E Q  4J2LC                 28    0.876 156.489   0.366   0.003   0.003   0.994

Explain the output.  Go back.
```

---
